# Exportin 1 (XPO1) inhibition leads to restoration of tumor suppressor miR-145 and consequent suppression of pancreatic cancer cell proliferation and migration

**DOI:** 10.18632/oncotarget.19285

**Published:** 2017-07-17

**Authors:** Asfar S. Azmi, Yiwei Li, Irfana Muqbil, Amro Aboukameel, William Senapedis, Erkan Baloglu, Yosef Landesman, Sharon Shacham, Michael G. Kauffman, Philip A. Philip, Ramzi M. Mohammad

**Affiliations:** ^1^ Department of Oncology, Barbara Ann Karmanos Cancer Institute, Wayne State University School of Medicine, Detroit, MI, USA; ^2^ Karyopharm Therapeutics Inc., Newton Centre, MA, USA

**Keywords:** XPO1, miR-145, pancreatic cancer, proliferation, migration

## Abstract

Pancreatic ductal adenocarcinoma (PDAC) is the third leading cause of cancer related deaths in the United States with a majority of these patients dying from aggressively invasive and metastatic disease. There is growing evidence that suggests an important role for microRNAs (miRNAs) in the pathobiology of aggressive PDAC. In this study, we found that the expression of miR-145 was significantly lower in PDAC cells when compared to normal pancreatic duct epithelial cells. Here we show that inhibition of the nuclear exporter protein exportin 1 (XPO1; also known as chromosome maintenance region 1 [CRM1]) by siRNA knockdown or by the Selective Inhibitor of Nuclear Export (SINE) compound (KPT-330; selinexor) increases miR-145 expression in PDAC cells resulting in the decreased cell proliferation and migration capacities. A similar result was obtained with forced expression of miR-145 in PDAC cells. To this end, SINE compound treatment mediated the down-regulation of known miR-145 targets genes including EGFR, MMP1, MT-MMP, c-Myc, Pak4 and Sox-2. In addition, selinexor induced the expression of two important tumor suppressive miRNAs miR-34c and let-7d leading to the up-regulation of p21^WAF1^. These results are the first to report that targeted inhibition of the nuclear export machinery could restore tumor suppressive miRNAs in PDAC that warrants further clinical investigations.

## INTRODUCTION

Pancreatic ductal adenocarcinoma (PDAC) is the third leading cause of cancer-related death in the United States with an estimated 53,000 new cases and 42,000 deaths in 2016 [1]. It is expected that PDAC will become the second leading cause of cancer-related death by 2030 [2]. For all stages combined, the 5-year survival of PDAC is only 7% with 2% for distant disease [3], resulting in an extremely poor prognosis and clinical outcome for patients with PDAC. The poor outcome and aggressiveness of PDAC could be caused by high instances of drug resistance and the invasive characteristic that develop during disease progression. Therefore, it is important to design new therapeutic strategies to inhibit proliferation, invasion and metastasis based on the novel molecular mechanism in order to successfully treat PDAC.

PDAC development is marked by alterations in several critical signal transduction proteins such as Ras, p53, EGFR and NF-κB. Therefore, targeting several molecular pathways could be more effective in the treatment of PDACs. The nuclear export protein XPO1 (also known as CRM1) regulates the localization of tumor suppressor proteins (TSPs). Cancer cell lines and patient samples from pancreatic, lung, breast, and other cancers have been shown to have an elevated level of XPO1 protein or mRNA and correlate with poor prognosis [4–10]. Aberrant XPO1 expression leads to increased nuclear export of TSPs away from their targets and perpetuation of the cancer phenotype. Selective Inhibitor of Nuclear Export (SINE) compound selinexor, binds specifically to XPO1 (as confirmed by crystallography and CRISPR/Cas9) and block cargoes such as TSPs from exiting the nucleus and leads to cell cycle arrest and genomic survey. Since XPO1 controls the cellular localization of multiple tumor suppressor proteins (TSPs) simultaneously, inhibition by selinexor blocks signal transduction pathways and results in cell cycle arrest, lack of cellular proliferation and induction of apoptosis in cancer while sparing normal cells. We have shown that SINE compounds can restore the function of multiple TSPs such as FOXO3a, p27, Par4 and p73, leading to PDAC cell death *in vitro* and tumor inhibition *in vivo* [7]. We also found that by nuclear retention of snail regulator FBXL5, selinexor reversed epithelial to mesenchymal transition (EMT) [4] which has been well known to induce drug resistance and cancer metastasis.

It is known that nuclear exporter proteins XPO1 and XPO5 mediate nucleo-cytoplasmic shuttling of mature microRNAs (miRNAs) [11–13]. MiRNAs are small non-coding RNAs that regulate gene expression and have garnered recent interest by cancer researchers. MiRNAs exert their gene-regulatory effects by binding to the 3’ untranslated region (3’UTR) of target mRNA, promote either degradation of the mRNA or inhibition of translation [14]. MiRNAs modulate various biological and pathological processes in cancer progression and development including aberrant cell growth, invasion and metastasis through manipulation of target gene expression [15]. More importantly, it has been found that aberrant up-regulation or down-regulation of specific miRNAs and their target genes in various cancer types including PDAC is associated with the development, progression and prognosis of those cancers [16, 17]. Therefore, miRNAs can be cancer oncogenes or tumor suppressors depending on their expression levels and/or cellular context [16]. Hence, it is important to design a therapeutic strategy that might modulate tumor suppressive or oncogenic miRNAs and be useful for the inhibition of PDAC progression, invasion and metastasis. In this study, we are the first to report that XPO1 inhibition (by RNAi or selinexor) can up-regulate the expression of tumor suppressive miR-145 and, in turn, down-regulate the expression of its target pathways, leading to the inhibition of proliferation and migration of PDAC cells.

## RESULTS

### Expression of miR-145 is lower in PDAC cells when compared with normal pancreatic duct epithelial cells

**Figure 1:**
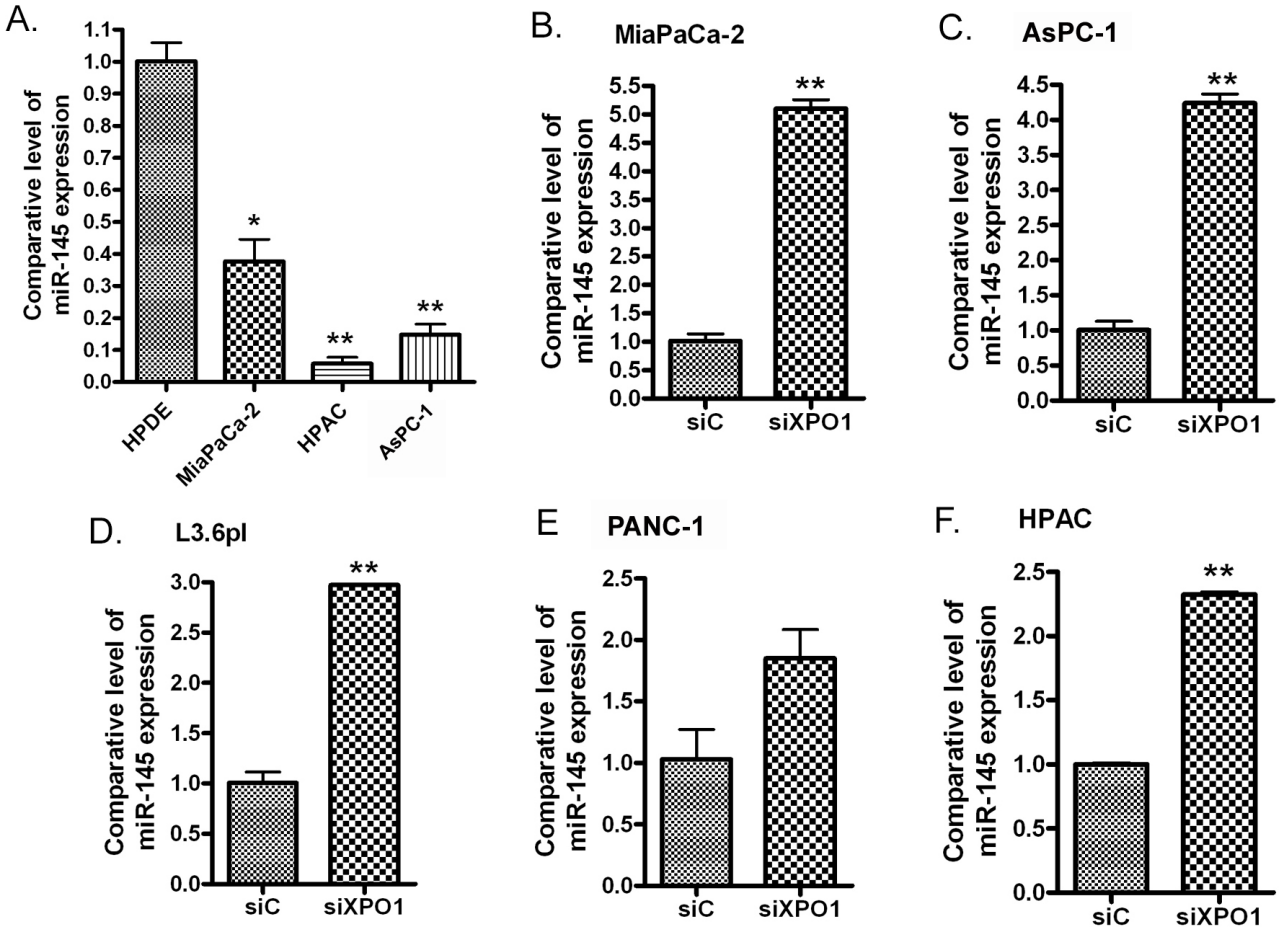
miR-145 was significantly down-regulated in PDAC cells **(A)** and transfection of XPO1 siRNA into PDAC cells induced the expression of miR-145. MiaPaCa-2 **(B)**, AsPC-1 **(C)**, L3.6pl **(D)**, PANC-1 **(E)** and HPAC **(F)** PDAC cells were transfected with XPO1 siRNA or control siRNA for 48 hours. The total RNAs from each sample were extracted and subjected to real-time RT-PCR for detection of miR-145 expression (*: p<0.05; **: p<0.01).

In order to investigate the expression of the tumor suppressive miRNA, miR-145, in PDAC and normal pancreatic duct epithelial (HPDE) cells, we utilized a miRNA array and miRNA real-time RT-PCR. The results from these assays showed that miR-145 expression in normal pancreatic HPDE cells was significantly higher when compared with MiaPaCa-2, AsPC-1, HPAC, PANC-1 and Colo357 PDAC cell lines (Table 1, Figure 1A). These results suggest that PDAC cells lose miR-145 expression during the carcinogenesis and cancer progression and may contribute to the aggressiveness of PDAC.

**Table 1:** Differential expression of miR-145 in PDAC cells measured by miRNA array

Cell lines	Normalized & averaged signal	StDev
HPDE	90.86	4.50
PANC-1	38.19	1.03
Colo357	1.83	0.13

### Treatment of PDAC cells with selinexor increases miR-145 expression through inhibition of XPO1 signaling

**Figure 2:**
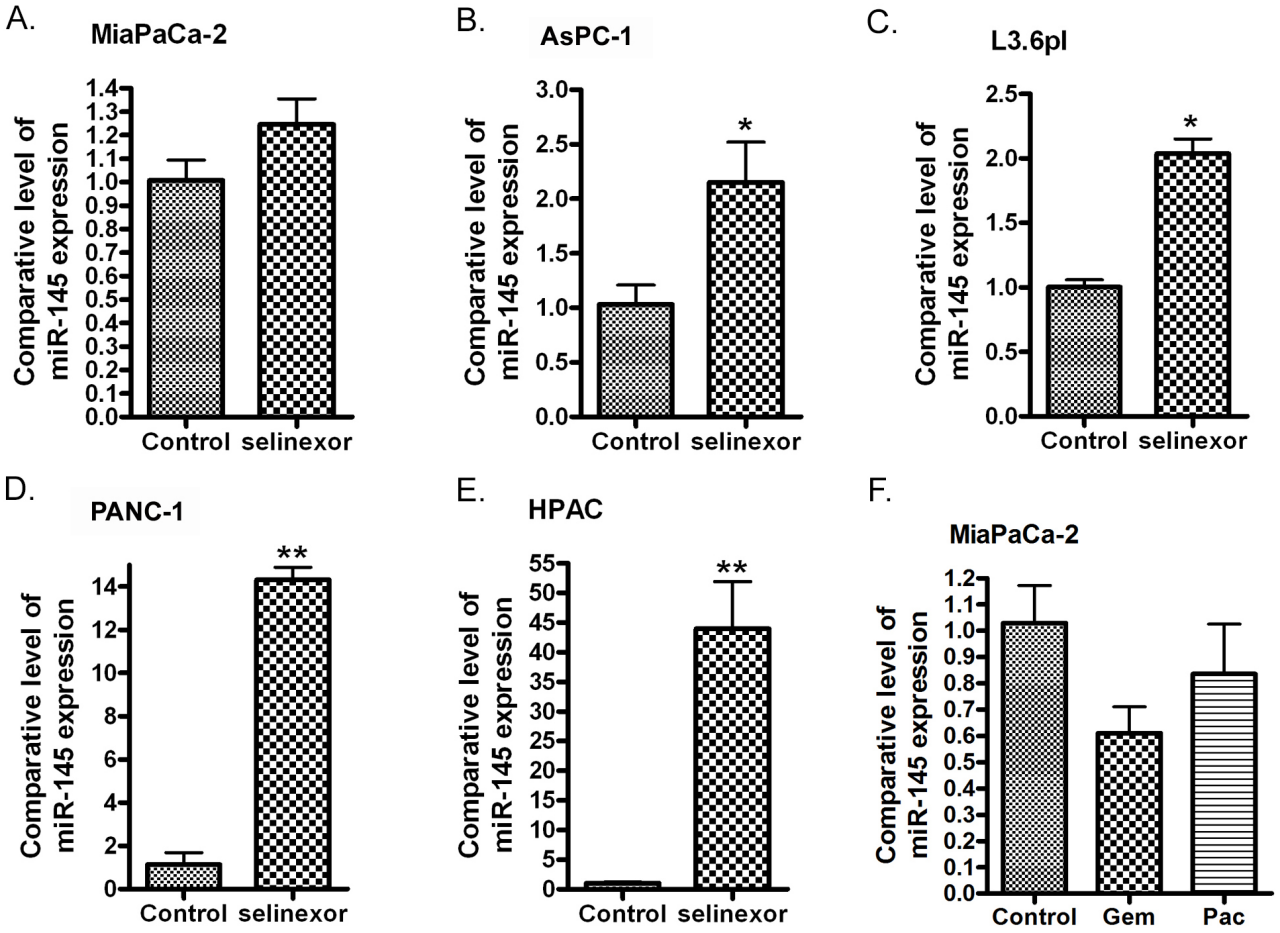
Treatment of PDAC cells with selinexor increased the expression of miR-145 MiaPaCa-2 **(A)**, AsPC-1 **(B)**, L3.6pl **(C)**, PANC-1 **(D)** and HPAC cells **(E)** were treated with 500 nM selinexor for 48 hours. MiaPaCa-2 cells were also treated with gemcitabine or paclitaxel for 48 hours. **(F)** (Gem: gemcitabine; Pac: paclitaxel). The total RNAs from each sample were extracted and subjected to real-time RT-PCR for detection of miR-145 expression (*: p<0.05; **: p<0.01).

In order to investigate the effects of XPO1 signaling on the expression of miRNAs, we transfected XPO1 siRNA into PDAC cells and tested the expression level of several tumor suppressive miRNAs. Interestingly, we found that the inhibition of XPO1 by siRNA increased the expression of miR-145 (Figure 1B-1F). More importantly, we found that the expression level of miR-145 expression was also significantly increased upon XPO1 inhibitor selinexor treatment in MiaPaCa-2, AsPC-1, L3.6pl, PANC-1 and HPAC PDAC cells (Figure 2A-2E), suggesting that selinexor increased miR-145 expression is mediated through XPO1 signal transduction. Since we have observed the growth inhibition of PDAC cells by selinexor [7], these results also suggest that the miR-145 induced by selinexor could be an inhibitory molecule for PDAC development and progression. Interestingly, we found that treatment with gemcitabine and paclitaxel, two conventional chemotherapeutics for PDAC, decreased the expression of miR-145 (Figure 2F), suggesting that down-regulation of miR-145 could be associated with drug resistance. Because miR-145 inhibits expression of target genes, we further tested the expression of miR-145 targets after re-expression of miR-145 or selinexor treatment in PDAC cells.

### Re-expression of miR-145 or treatment with selinexor results in inhibition of miR-145 targets and signaling pathways in PDAC cells

**Figure 3:**
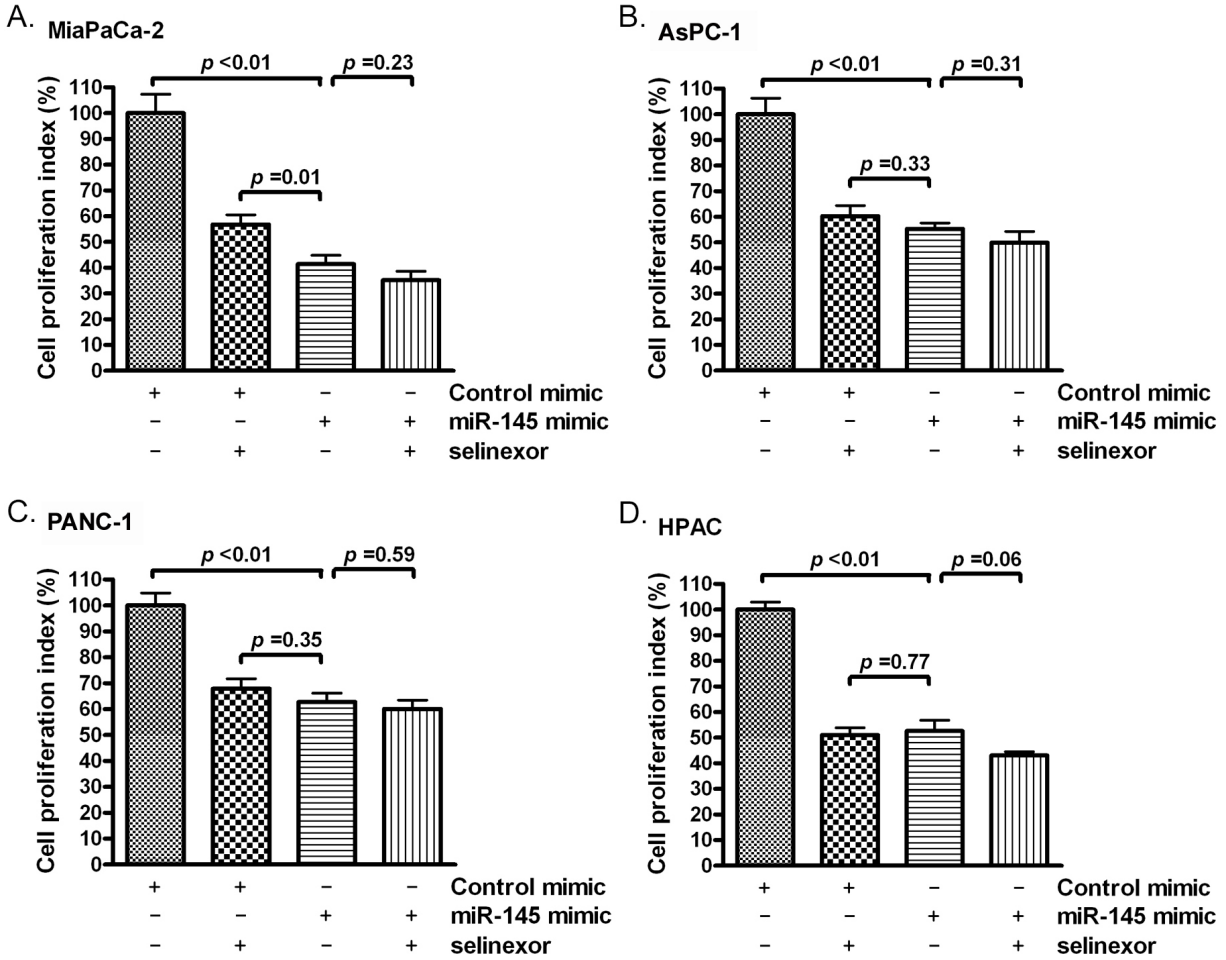
Selinexor treatment or miR-145 mimic transfection inhibited the proliferation of PDAC cells MiaPaCa-2 **(A)**, AsPC-1 **(B)**, PANC-1 **(C)** and HPAC **(D)** cells were treated with 500 nM selinexor or transfected with miR-145 mimic or control mimic. The cell proliferation index was measured by MTT assay as described in the section of “Materials and Methods”.

We measured cell proliferation in PDAC after miR-145 mimic or selinexor treatment. We found that the miR-145 mimic or selinexor treatment inhibited proliferation rate of PDAC cells (Figure 3A-3D). These results suggest that inhibition of cellular proliferation by selinexor is at least partially controlled through induction of miR-145 expression.

**Figure 4:**
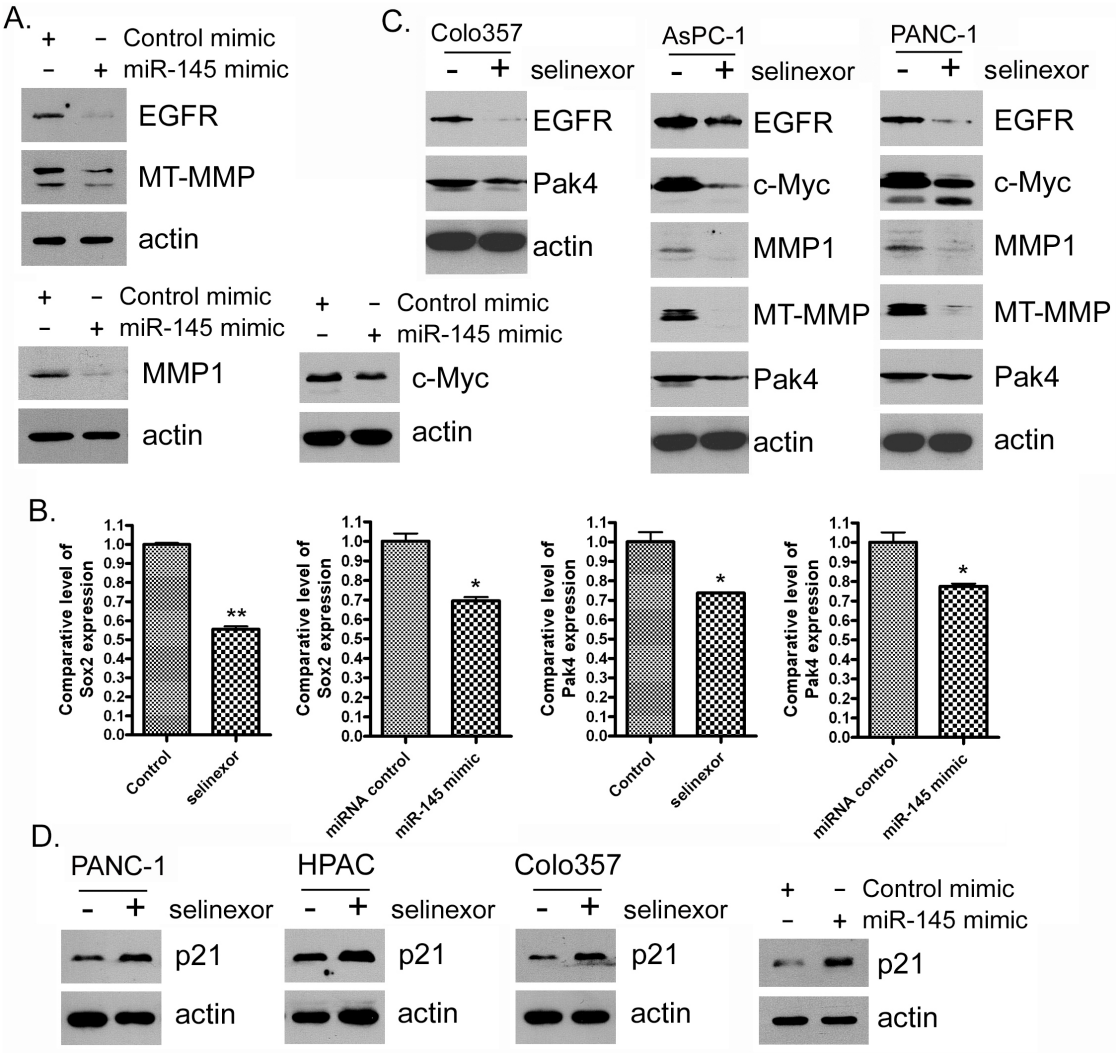
Selinexor treatment or miR-145 mimic transfection inhibited the expression of miR-145 target or downstream genes at protein or RNA level **(A-D)** MiaPaCa-2, AsPC-1, PANC-1, Colo357 and HPAC cells were treated with 500nM selinexor or transfected with miR-145 mimic or control mimic for 72 hours. Total protein was extracted from each sample and subjected to Western Blot analysis for detection of EGFR, MMP1, MT-MMP, c-Myc, Pak4 and p21^WAF1^ expression at protein level (A, C and D). Total RNA was extracted and subjected to real-time PCR for detection of Sox-2 and Pak4 (B).

In order to understand whether selinexor can regulate specific signaling pathways through the up-regulation of miR-145, we transfected PDAC cells with a miR-145 mimic or treated the cells with selinexor. We found that the introduction of miR-145 mimic or selinexor treatment of PDAC cells resulted in a down-regulation of miR-145 targets and signaling pathways including EGFR, MMP1, MT-MMP, c-Myc, PAK4 and Sox2 mRNA or protein (Figure 4A-4C). These results suggest that the inhibitory effect selinexor has on cellular signaling pathways is at least partially mediated through the induction of miR-145 expression. In addition, miR-145 transfection and selinexor treatment can also induce the expression of cell cycle inhibitor, p21^WAF1^ (Figure 4D). Because these miR-145 targets control cell proliferation and migration, we next investigated whether the miR-145 mimic or selinexor treatment has an effect on these cellular processes in PDAC cells.

### miR-145 transfection or selinexor treatment inhibited PDAC cell migration

**Figure 5:**
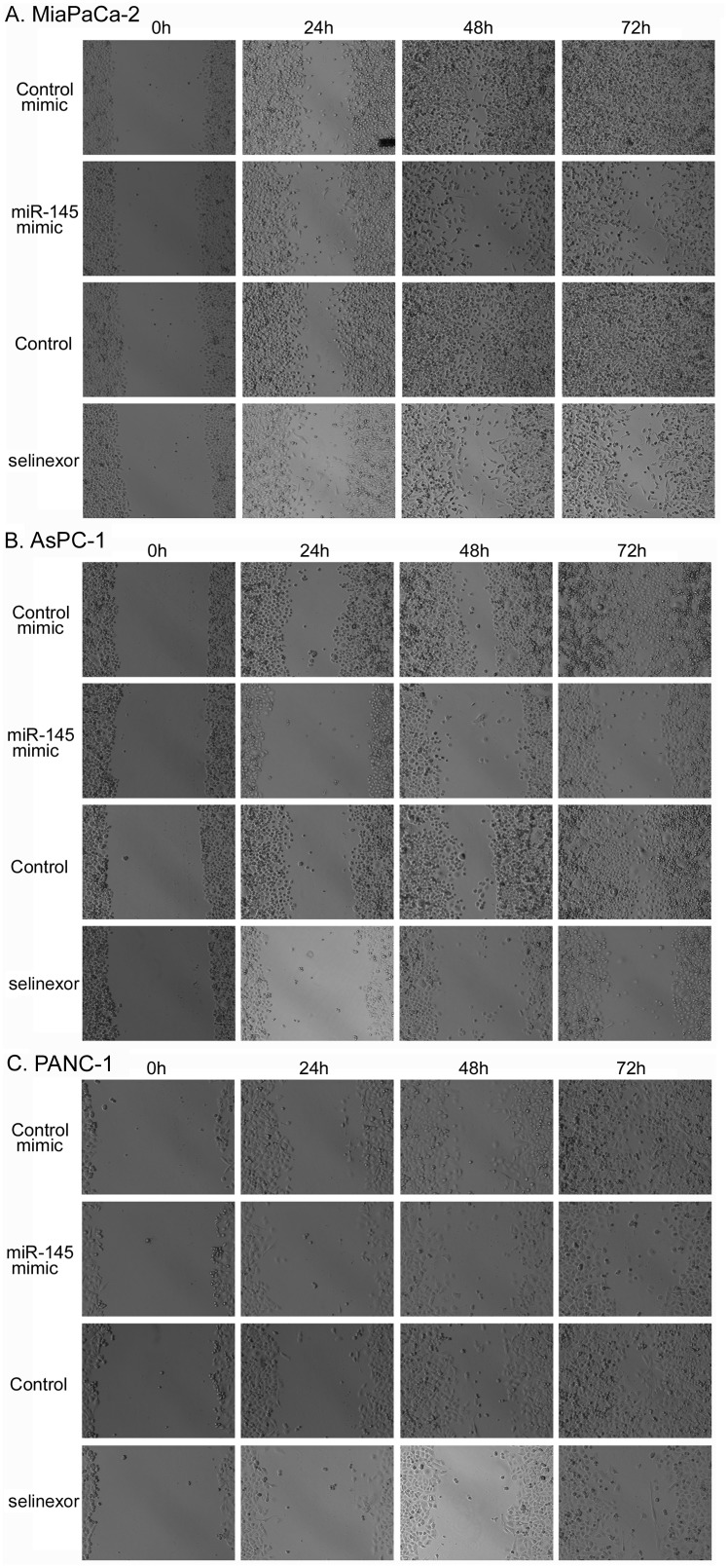
Selinexor treatment or miR-145 mimic transfection suppressed the migration activity of PDAC cells Selinexor treated or miR-145 mimic transfected MiaPaCa-2 **(A)**, AsPC-1 **(B)** and PANC-1 **(C)** PDAC cells were seeded in 6 well plate and subjected to wound healing assay for 3 days as described in the section of “Materials and Methods”. The cells were photographed in each day.

We employed a traditional scratch assay of PDAC cell culture to determine whether miR-145 can control cell migration. By measuring wound closure, we found that PDAC cell migration was significantly inhibited by miR-145 mimic or selinexor treatment (Figure 5A-5C). Since we observed that selinexor treatment inhibited the expression of miR-145 targets controlling cell migration, these results suggest that selinexor treatment could inhibit PDAC cell migration, in part, through up-regulation of miR-145 expression and down-regulation of miR-145 targets such as EGFR, MMP-1 and MT-MMP.

### Impact of selinexor on additional PDAC related miRNAs

**Figure 6:**
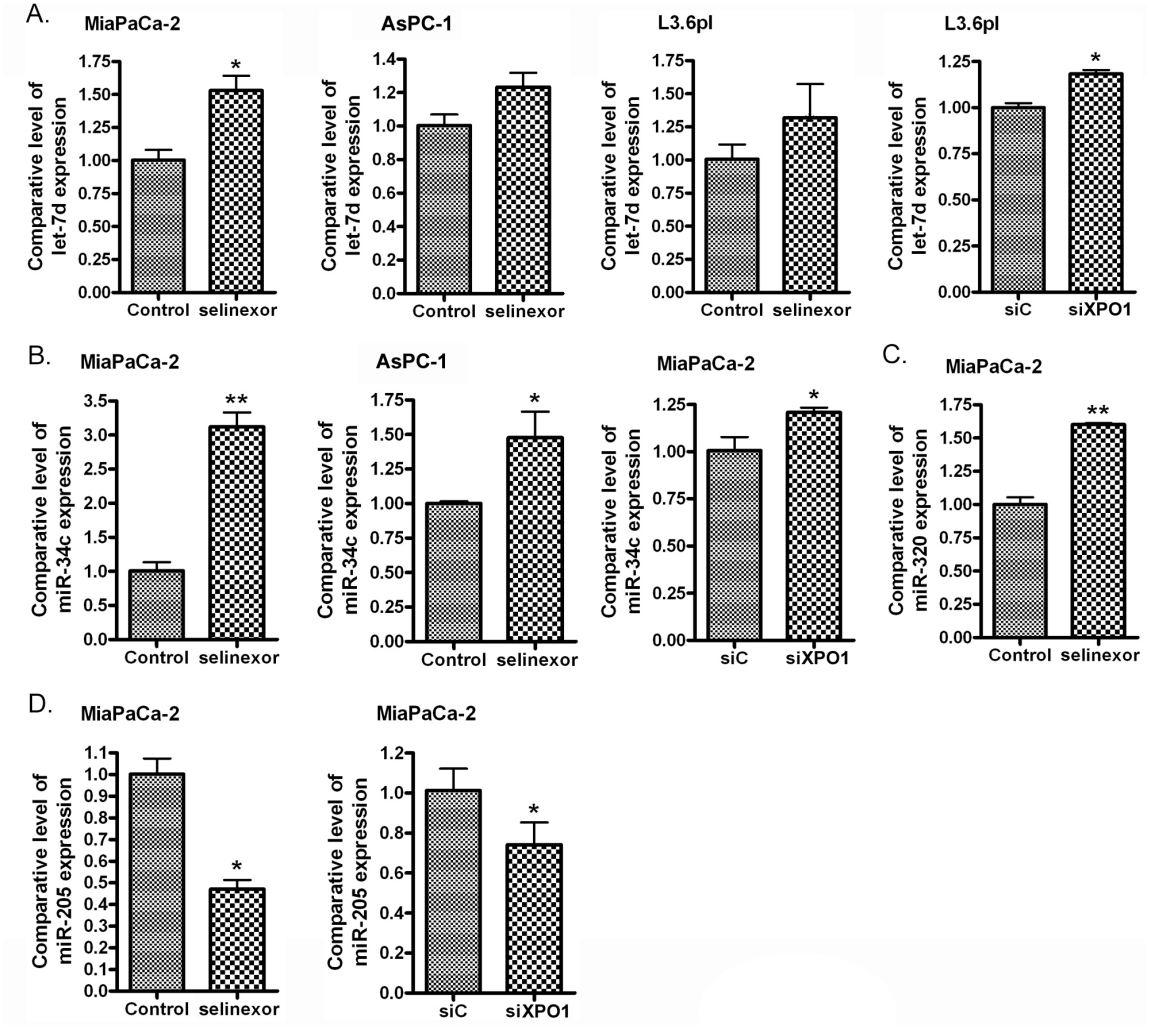
Selinexor treatment or XPO1 siRNA transfection induced the expression of let-7d **(A)**, miR-34c **(B)** and miR-320 **(C)**, and reduced the expression of miR-205 **(D)**. MiaPaCa-2, AsPC-1 and L3.6pl PDAC cells were treated with 500 nM selinexor or transfected with miR-145 mimic or control mimic for 48 hours. The total RNA from each sample was extracted and subjected to real-time RT-PCR for detection of let-7d, miR-34c, miR-320 and miR-205 expression.

In addition to the induction of miR-145 in PDAC cells, RNAi of XPO1 or selinexor treatment increased the expression of other tumor suppressive miRNAs including let-7d, miR-34c and miR-320 (Figure 6A-6C), and decreased the expression of oncogenic miR-205 (Figure 6D). These results show that inhibition of XPO1 through either RNAi or selinexor treatment may decrease PDAC development and progression through modulation of multiple miRNAs.

## DISCUSSION

This is the first report that XPO1 inhibition can modulate a critical tumor suppressive miRNA (miR-145) that may control PDAC cell proliferation, invasiveness and metastasis. MiRNAs play an important role in the regulation of a large amount of target genes that control carcinogenesis and cancer progression. Therefore, detecting aberrant expression of miRNAs in different tumors types using miRNA microarray or RT-PCR was used to elucidate the molecular regulations of oncogenic and tumor suppressive signaling controlled by mRNA expression [17–19]. Since down-regulation of miR-145 has been detected in leukemia, prostate, colon, ovarian, hepatic, pancreatic and other cancers [16, 20–24]; it has been recognized as an important tumor suppressive miRNA. MiR-145 expression is lower in PDAC cells when compared to normal HPDE while its re-expression in PDAC results in suppression of cell proliferation, clonogenicity, migration and invasion through the regulation of different cell signaling pathways [25–27]. In addition, miR-145 re-expression inhibits cancer cell proliferation, invasion and metastasis in breast, lung, colon, gastric, and ovarian cancers [28–32]. Thus, any treatment strategy which increases the miR-145 expression could be beneficial for many different types of cancer. In our study, we found lower miR-145 expression in PDAC cells when compared to normal pancreatic duct epithelial cells (HPDE cells), which is consistent with the report by other investigators showing lower expression of miR-145 in pancreatic cancer tissues [22]. More importantly, we found that XPO1 RNAi or SINE compound (selinexor) treatment could up-regulated miR-145 expression.

We discovered that exposure of PDAC cells to selinexor or miR-145 mimic can reduce cell proliferation and migration. Considering that selinexor can up-regulate miR-145, our results suggest that the inhibition of PDAC cell proliferation and migration is partially mediated by miR-145-regulated signaling transduction. Our findings are consistent with recent reports that show up-regulation of miR-145 inhibits cancer cell growth, migration and metastasis in breast, hepatic and esophageal cancers [33–35]. Collectively, we demonstrate that selinexor could be a potent agent for the suppression of PDAC proliferation, migration, invasion and metastasis through the up-regulation of tumor suppressive miR-145. This result could be used for designing novel therapeutic strategy in combination with other standard therapeutic agents.

Previously we and others demonstrated that selinexor inhibited cell growth and induced apoptosis [4, 7, 36–38]. Our work in different PDAC models led to a Phase Ib/II clinical trial of selinexor in combination with gemcitabine and nab-paclitaxel for the treatment of metastatic pancreatic cancer (ClinicalTrials.Gov identifier NCT02178436 https://clinicaltrials.gov/ct2/show/NCT02178436). Building our translational findings, we evaluated the molecular mechanisms of PDAC progression induced by miRNA regulation after selinexor treatment. We found that selinexor treatment inhibited the expression of miR-145 targets which include but are not limited to EGFR, MMP1, MT-MMP, c-Myc, Pak4 and Sox2. These results suggest that cell proliferation, migration and invasion repressed by selinexor treatment are mediated through the up-regulation of miR-145 expression and subsequent down-regulation target genes such as MMP1, MT-MMP, and EGFR [30, 39–42]. In addition, Sox2, PAK4 and c-Myc are regulators of cancer cell proliferation [43–45]. Sox2 is a predicted target of miR-145 [46] while PAK4 and c-Myc are confirmed miR-145 targets [47, 48]. Taken together we propose that selinexor treatment increases miR-145 expression and, in turn, inhibits expression of its target genes such as, Sox2, Pak4 and c-Myc, leading to inhibition of PDAC cell proliferation.

The let-7 and miR-34 miRNA families are down-regulated in PDAC [49, 50]. Studies found that both let-7 and miR-34 could contribute to the up-regulation of p21^WAF1^, leading to cell cycle arrest and inhibition of cell proliferation [51, 52]. In addition, selinexor has been found to increase the expression and nuclear accumulation of p53 [53, 54] which, in turn, induces p21^WAF1^. Therefore, the inhibition of cell proliferation by selinexor could also be mediated through miRNA or p53-induced expression of p21^WAF1^. However, further in-depth mechanistic studies are warranted to understand the impact selinexor treatment has on miRNA expression, cell proliferation and migration. It is well known that XPO1 controls the protein shuttling from nucleus to cytosol. However, how XPO1 controls miRNA shuttling and subsequently increases miR-145 and other tumor suppressive miRNAs is still poorly understood. This could be mediated through the regulation of p53 pathway since XPO1 inhibition can increase total p53 as well as increase its nuclear localization [53, 54]. The transactivation of p53 up-regulates the expression of miR-145 and miR-34 [55, 56]. Therefore, the up-regulation of miR-145 and other tumor suppressive miRNAs after selinexor treatment could be mediated through the nuclear retention and activation of p53. We are currently conducting additional studies to elucidate how selinexor increases the expression of miR-145 and other tumor suppressive miRNAs.

Previously, we conducted pre-clinical anti-tumor efficacy studies of selinexor either as a single agent or in combination with gemcitabine and nab-paclitaxel [7, 57, 58]. We are evaluating residual tumors from our previous studies for re-activation of miR-145 and suppression of other oncomirs. We are also performing *in vivo* studies to evaluate the impact of miR-145 knockdown on MiaPaCa-2 tumor growth in mice. These on-going studies which involve multiple transfections of cells with miR-145 prior to implantation in mice at a sub-cutaneous and orthotopic site, will be included in future reports. Aside from these cellular and animal model studies, our additional investigations include the analysis of miRNA expression changes in patient tissue specimen from the selinexor-gemcitabine-nab-pactlitaxel Phase Ib/II study (NCT02178436). Such studies are anticipated to validate our *in vitro* and *ex vivo* findings and may help the design of future clinical studies with selinexor in PDAC patients that have been stratified for selected miRNAs.

In conclusion, we clearly demonstrate that targeting the nuclear export machinery especially XPO1 up-regulates miR-145, miR-34 and let-7 expression which are lower in PDAC cells when compared to normal HPDE cells. The up-regulation of miR-145 by selinexor (a Phase II inhibitor of XPO1) inhibits PDAC cell proliferation and migration through down-regulation of miR-145 target genes such as MMP1, MT-MMP, EGFR, Sox2, c-Myc, and PAK4. Additional *in vivo* studies and clinical trials are needed to evaluate whether selinexor could be used in combination with conventional chemotherapeutics for the better treatment of PDAC mediated through up-regulation of tumor suppressive miRNAs.

## MATERIALS AND METHODS

### Cell lines, reagents, and antibodies

MiaPaCa-2, HPAC, AsPC-1, and PANC-1 PDAC cells were purchased from American Type Culture Collection (ATCC, Manassas, VA) and maintained in DMEM (Invitrogen, Carlsbad, CA) supplemented with 10% fetal bovine serum (FBS), 100 U/mL penicillin and 100 μg/mL streptomycin in a 5% CO_2_ atmosphere at 37°C. Colo357 and L3.6pl PDAC cells and human pancreatic duct epithelial (HPDE) cells were obtained from MD Anderson Cancer Center and cultured in DMEM/FBS or keratinocyte serum-free medium supplied with 5 ng/mL of epidermal growth factor and 50 μg/mL of bovine pituitary extract (Invitrogen). The cell lines have been tested and authenticated in core facility Applied Genomics Technology Center at Wayne State University. The method used for testing was short tandem repeat (STR) profiling using the PowerPlex^®^ 16 System from Promega (Madison, WI). XPO1 inhibitor selinexor (Karyopharm Therapeutics, Newton, MA) was dissolved in DMSO to make a 1 mM stock solution. Anti-EGFR (Santa Cruz, Santa Cruz, CA), anti-c-Myc (Cell Signaling, Danvers, MA), anti-MT-MMP (Cell Signaling), anti-MMP1 (R&D Biosystems, Minneapolis, MN), anti-Pak4 (Santa Cruz), anti-p21 (Millipore, Billerica, MA), and anti-β-actin (Sigma, St. Louis, MO) primary antibodies were used for Western Blot analysis.

### RNA isolation and miRNA real-time RT-PCR

Total RNA was extracted and purified by using the miRNeasy Mini Kit and RNase-free DNase Set (QIAGEN, Valencia, CA) following the protocol provided by the manufacturer. The expression level of miR-145, miR-34c, let-7d, miR-320 and miR-205 in KPT-330 treated or un-treated and control siRNA or XPO1 siRNA transfected PDAC cells was analyzed by using Universal cDNA Synthesis Kit (Exiqon, Woburn, MA), specific LNA™ PCR primer set (Exiqon), and SYBR Green RT-PCR Reagents (Applied biosystems). The PCR program was initiated by 10 min at 95°C before 40 thermal cycles, each of 15 s at 95°C and 1 min at 60°C. Data were analyzed according to the comparative Ct method and were normalized by RNU44 and RNU1a1 expression in each sample.

### miRNA array and data analysis

Five micrograms of each total RNA sample from HPDE, Colo357, and PANC-1 cells were sent to a service provider for completion of the miRNA arrays (LCSciences, Houston, TX). In LCSciences, the total RNA samples were enriched for miRNAs and the miRNA arrays were performed on μParaFlo™ microfluidic chips (version 10.0), each of which has a miRNA probe region with multiple repeat regions that detect 711 miRNAs. Multiple control probes are also included on the arrays for assessing various chip and assay qualities such as uniformity and specificity. Chips were scanned and the signal intensity data was obtained. Then, the data was analyzed by subtracting the background and normalizing the signals to balance the intensities of transcripts so that differential expression ratios can be compared and calculated.

### mRNA real-time RT-PCR

The expression level of Sox2 and Pak4 in KPT-330 treated or un-treated and miR-control mimic or miR-145 mimic transfected PDAC cells was analyzed by real-time RT-PCR using High Capacity cDNA Reverse Transcription Kit and SYBR Green Master Mixture from Applied Biosystems. The sequences of primers used were: Sox2-F: ACATGAACGGCTGGAGCAA; Sox2-R: GTAGGACATGCTGTAGGTGGG; Pak4-F: GTGCAAGAGAGCTGAGGGAG; Pak4-R: ATGCTGGTGGGACAGAAGTG; GAPDH-F: CCACATCGCTCAGACACCAT; GAPDH-R: ACCAGAGTTAAAAGCAGCCCT; 18S-F: GCAATTATTCCCCATGAACG; and 18S-R: GGCCTCACTAAACCATCCAA. The PCR was initiated by 10 min at 95°C before 40 thermal cycles, each of 15 s at 95°C and 1 min at 60°C. Data were analyzed according to the comparative Ct method and were normalized by GAPDH and 18S rRNA expression in each sample.

### Western blot analysis

Western Blot analysis was conducted to measure the alterations in the protein expression of genes, which are targets or downstream genes of miR-145. MiaPaCa-2, AsPC-1, HPAC and PANC-1 PC cells were treated with or without 300-500nM selinexor for 72 hours. In a separated experiment, these cells were transfected with miR-control or miR-145 mimic for 72 hours. After treatment or transfection, the cells were lysed in RIPA buffer, and protein concentration was measured using BCA protein assay (PIERCE, Rockford, IL). The proteins were subjected to 10% or 14% SDS-PAGE, and electrophoretically transferred to nitrocellulose membrane. The membranes were incubated with specific primary antibodies, and subsequently incubated with secondary antibody conjugated with peroxidase (Bio-rad, Hercules, CA). The signal was detected using the chemiluminescent detection system (PIERCE).

### Re-expression of miR-145 in PDAC cells

MiaPaCa-2, AsPC-1, HPAC and PANC-1 PDAC cells were seeded in 6 well plates and transfected with miR-control or miR-145 mimic (Applied biosystems) at a final concentration of 20 nM using DharmaFact Transfection Reagent (Dharmacon, Lafayette. CO). After 3 days of transfection, the cells were split and transfected repeatedly with the miRNA mimic or control every 3-4 days for indicated times. Total RNA from each samples were then extracted. One microgram of RNA was subject to RT-PCR using the High Capacity cDNA Reverse Transcription Kit (Applied Biosystems) and SYBR Green PCR Master Mix (Applied Biosystems) as described earlier. Total proteins from each sample were also extracted and subject to Western Blot analysis as described earlier.

### Inhibition of XPO1 expression by siRNA in PDAC cells

MiaPaCa-2 and L3.6pl, AsPC-1 and PANC-1 cells were seeded in a 6 well plate (1.2X10^5^ cells per well) and incubated at 37°C for 24 hours. The cells were then transfected with XPO1 siRNA (Santa Cruz) or control siRNA by DharmaFact Transfection Reagent (Dharmacon) for 72 hours. Then, the RNA was extracted and subject to miRNA RT-PCR.

### Wound healing assay

Wound healing assay was conducted to study the effects of selinexor and miR-145 mimic on the migration of PDAC cells. miR-145 mimic or miR-control mimic transfected or un-transfected MiaPaCa-2, AsPC-1 and PANC-1 cells were seeded into 6 well plate at high density and cultured until sub-confluent. A yellow pipette tip was used to make a straight scratch, simulating a wound. The cells were washed with medium and the detached cells were removed. Un-transfected cells were treated with 500nM selinexor. The cells were cultured for additional 72 hours with changing medium and treatments for every day. The scratched cell images were taken every day under an EVOS microscope.

### Growth inhibition assay

MiaPaCa-2, PANC-1, AsPC-1 and HPAC cells were transfected with miR-145 or miR-control mimic for 9 days as described. Then, the transfected cells were seeded in 96 well plates. Un-transfected cells were also seeded in 96 well plate and treated with 500nM selinexor for 3 days. After three days, the cells were subjected to cell proliferation assay using MTT [3-(4,5-dimethylthiazol-2-yl)-2,5-diphenyltetrazolium bromide]. The spectrophotometric absorbance of the samples was determined by using a plate reader SynergyHT (BioTek, Winooski, WI) at 570 nm.

### Statistics

Wherever appropriate, the data were subjected to a Student’s t-test using GraphPad Prism software. p < 0.05 was considered statistically significant.
